# HIF-1*α*–Nrf2 crosstalk in myocardial ischemia–reperfusion injury: a potential preconditioning-responsive protective network

**DOI:** 10.3389/fmed.2026.1976117

**Published:** 2026-10-15

**Authors:** Xiaoqiang Guo, Chaojie Wang, Shuyan Zhang, Mengqi Li, Chen Chen

**Affiliations:** 1 Acupuncture and Massage College, Graduate School, Heilongjiang University of Chinese Medicine, Harbin, China; 2 Liaoning University of Chinese Medicine, Shenyang, China; 3 The Second Affiliated Hospital of Heilongjiang University of Chinese Medicine, Harbin, China

**Keywords:** cardioprotection, HIF-1*α*, molecular crosstalk, myocardial ischemia–reperfusion injury, Nrf2, oxidative stress, preconditioning

## Abstract

Myocardial ischemia–reperfusion injury (MIRI) is a dynamic pathological process in which metabolic and mitochondrial disturbances during ischemia predispose the myocardium to oxidative damage upon reperfusion. This temporal coupling poses a fundamental limitation to single-pathway cardioprotection, as interventions directed at one stage of injury may fail to adequately address the evolving pathophysiological cascade. Hypoxia-inducible factor-1*α* (HIF-1*α*) and nuclear factor erythroid 2-related factor 2 (Nrf2), key regulators of hypoxic adaptation and cellular redox homeostasis, respectively, may constitute an interconnected protective network that spans the ischemia–reperfusion transition. HIF-1*α* promotes metabolic reprogramming and mitochondrial adaptation during ischemia, thereby reducing oxidative susceptibility, whereas Nrf2 enhances glutathione metabolism, peroxide detoxification, and resistance to lipid peroxidation during reperfusion. Emerging evidence further suggests the possibility of reciprocal crosstalk between these pathways, with HIF-1*α* reported to facilitate Nrf2 activation and Nrf2 to support HIF-1*α* transcriptional availability and protein stability. This coupling may enable preconditioning to establish metabolic and mitochondrial adaptation before ischemia while simultaneously building an antioxidant defense reserve for reperfusion, while sustained metabolic adaptation during early reperfusion provides transitional support for the recovery of oxidative metabolism, thereby limiting secondary oxidative injury. Compared with isolated pathway activation, coordinated HIF-1*α*–Nrf2 regulation may therefore provide broader and more temporally integrated cardioprotection by simultaneously reducing oxidative susceptibility and enhancing resistance to reperfusion stress. Pharmacological and non-pharmacological preconditioning studies further support the therapeutic potential of this network-based strategy, highlighting HIF-1*α*–Nrf2 crosstalk as a promising framework for optimizing cardioprotection in MIRI.

## 1 Introduction

Myocardial ischemia-reperfusion injury (MIRI) is a major pathological process that limits the clinical benefit of reperfusion therapy (1). Its pathological basis does not begin with the burst of reactive oxygen species (ROS) after restoration of blood flow; rather, it develops progressively during ischemia (2, 3). Suppressed oxidative phosphorylation, accumulation of metabolic intermediates, and mitochondrial dysfunction render the myocardium highly vulnerable to reoxygenation. The abrupt restoration of oxygen supply during reperfusion rapidly converts this vulnerable state into lipid peroxidation, mitochondrial permeability transition pore opening, disruption of calcium homeostasis, and cardiomyocyte death (4, 5). Thus, MIRI is fundamentally a temporally ordered pathological process in which metabolic and mitochondrial disturbances during ischemia are followed continuously by oxidative injury during reperfusion.

This temporal sequence explains why interventions directed only at post-reperfusion injury often provide incomplete protection (6, 7). ROS levels can rise within a very short period during early reperfusion, whereas transcription-dependent endogenous protective programs require protein stabilization, transcriptional activation, and accumulation of effector molecules and therefore may not be established rapidly enough to cover the initial injury window. Moreover, scavenging ROS after reperfusion cannot reverse the metabolic and mitochondrial abnormalities that have already developed during ischemia (8, 9). Accordingly, shifting the intervention window earlier and using preconditioning to recruit endogenous adaptive mechanisms before severe ischemia and reperfusion, while simultaneously improving the metabolic state during ischemia and establishing antioxidant reserves for reperfusion, is more consistent with the pathological kinetics of MIRI (10).

HIF-1*α* and Nrf2 are key regulatory nodes of hypoxic adaptation and redox defense, respectively (11, 12). HIF-1*α* coordinates metabolic and mitochondrial adaptation during ischemia, whereas Nrf2 provides antioxidant defense for reperfusion (13, 14). Emerging evidence further suggests reciprocal regulation between these factors: HIF-1*α* can promote Nrf2 expression and transcriptional activation, whereas Nrf2 can support hypoxic adaptation by modulating *HIF1A* transcriptional availability and HIF-1*α* protein homeostasis (15–17). HIF-1*α* and Nrf2 may therefore form a mutually supportive regulatory network that links metabolic and mitochondrial adaptation during ischemia to antioxidant defense during reperfusion; however, the stability and therapeutic controllability of this network in MIRI remain to be established.

Although the individual cardioprotective roles of HIF-1*α* and Nrf2 in MIRI have been extensively investigated, their bidirectional regulation, responsiveness to preconditioning, and integrated protective value have not been systematically synthesized (18–20). This review uses the temporal pathophysiology of MIRI as its organizing framework to summarize the endogenous protective functions mediated by HIF-1*α* and Nrf2, examine the molecular basis of their reciprocal regulation, and integrate current evidence on pharmacological preconditioning and non-pharmacological preadaptation of this network. On this basis, we further evaluate the biological plausibility, therapeutic potential, and evidentiary boundaries of the HIF-1*α*-Nrf2 network as a candidate target in MIRI, providing a theoretical basis for moving cardioprotective strategies from single-pathway activation toward network-based and temporally coordinated regulation.

## 2 Literature search strategy

This narrative review used a structured literature search to obtain a broad and balanced overview of the available evidence. PubMed, Scopus and Web of Science were searched from database inception to July 2026 using combinations of terms related to myocardial ischemia-reperfusion injury (MIRI), HIF-1*α*/*HIF1A*, Nrf2/NFE2L2, oxidative stress, preconditioning, remote ischemic conditioning, and preadaptation. Boolean operators (‘AND’ and ‘OR’) were used to combine search concepts, and reference lists of relevant reviews and key original studies were additionally screened to identify potentially relevant publications.

Original experimental studies, clinical studies, and selected reviews were considered when they contributed to the major themes of this review, including MIRI pathophysiology, HIF-1*α*-mediated hypoxic and metabolic adaptation, Nrf2-mediated antioxidant defense, HIF-1*α*–Nrf2 crosstalk, and preconditioning-related interventions. Priority was given to myocardial I/R, cardiomyocyte, and cardiac preconditioning studies. Evidence from other cardiac conditions or non-cardiac hypoxia, I/R, H/R, OGD/R, and redox-stress models was retained when it provided mechanistically informative evidence that could not be adequately addressed by available MIRI studies.

For studies addressing HIF-1*α*–Nrf2 regulation, greater weight was given to experiments incorporating genetic manipulation, pharmacological blockade, functional rescue, transcriptional assays, protein-stability analysis, or nuclear-translocation measurements rather than studies showing only parallel changes in pathway expression. Representative publications were selected according to mechanistic relevance, methodological quality, recency, and their contribution to the conceptual framework of this review.

## 3 Oxidative stress

### 3.1 Temporal evolution of oxidative stress

Oxidative stress is a major pathological driver of MIRI and evolves in a distinct yet continuous stage-dependent manner (21, 22). Because the myocardium relies heavily on mitochondrial oxidative phosphorylation for energy production, it is particularly sensitive to disturbances in redox homeostasis. During ischemia, mitochondrial oxidative phosphorylation is suppressed, and cardiomyocytes shift toward anaerobic glycolysis to maintain limited adenosine triphosphate (ATP) production. This is accompanied by progressive acidosis, disruption of Na+/Ca2 + homeostasis, loss of mitochondrial membrane potential, and respiratory-chain dysfunction (23, 24). Because oxygen availability is restricted, ischemia generally does not produce the high-flux ROS burst characteristic of reperfusion. Nevertheless, sustained energetic derangement and mitochondrial injury substantially alter the cellular redox state, progressively creating an oxidative vulnerability that renders the myocardium highly sensitive to reoxygenation (25).

This vulnerable state is shaped by the accumulation of metabolic substrates, alterations in pro-oxidant enzyme systems, and mitochondrial injury (26–28). During ischemia, continued ATP degradation leads to hypoxanthine accumulation and is accompanied by conversion of xanthine dehydrogenase to xanthine oxidase, whereas interruption of the tricarboxylic acid cycle and respiratory-chain dysfunction promotes the accumulation of reduced metabolites such as succinate (29, 30). Concurrently, acidosis, Ca2 + overload, membrane-potential disturbances, and damage to respiratory-chain complexes increase the proportion of dysfunctional mitochondria. Although these changes may not immediately cause extensive oxidative damage during ischemia, they establish the metabolic substrates, enzymatic conditions, and organelle abnormalities required for rapid ROS generation upon reoxygenation.

Reperfusion reintroduces oxygen into a cellular environment in which metabolic and mitochondrial homeostasis has already been compromised, thereby activating multiple ROS-generating pathways almost simultaneously (31). Succinate accumulated during ischemia can be rapidly oxidized after reoxygenation, driving electrons backward through the coenzyme Q pool toward complex I, producing reverse electron transport (RET) and large amounts of superoxide (32). Xanthine oxidase uses the restored oxygen supply to metabolize hypoxanthine and xanthine, further generating superoxide and hydrogen peroxide. Inflammatory mediators, vasoactive factors, and mechanical stress can additionally activate nicotinamide adenine dinucleotide phosphate oxidases (NOX), whereas uncoupling of endothelial nitric oxide synthase (eNOS) increases superoxide production while reducing nitric oxide bioavailability (33–36). The coordinated release of ROS from these different sources collectively drives the oxidative burst of early reperfusion (37).

When the rate of ROS generation exceeds the processing capacity of endogenous antioxidant systems, redox imbalance progresses from a biochemical disturbance to structural and functional injury (38–40). Lipid peroxidation disrupts the integrity of cellular and mitochondrial membranes and increases susceptibility to iron-dependent lipid peroxidation and associated forms of cell death (39). Oxidative modification of proteins impairs metabolic enzymes, respiratory-chain complexes, and ion-transport proteins (41), whereas oxidative DNA damage further activates damage-response and cell-death programs (38, 42). At the mitochondrial level, ROS and Ca2 + overload act synergistically to promote opening of the mitochondrial permeability transition pore (mPTP), leading to loss of membrane potential, further ATP depletion, and cytochrome c release (38, 41). Oxidative stress also amplifies inflammation, endothelial dysfunction, and microcirculatory injury; these secondary changes further damage mitochondria and promote ROS production, creating a self-sustaining injury-amplification loop (38, 42).

Taken together, oxidative injury during reperfusion is not triggered by reoxygenation alone but represents the concentrated manifestation of metabolic and mitochondrial abnormalities established during ischemia once oxygen supply is restored. The ischemic phase largely determines myocardial oxidative susceptibility at reoxygenation, whereas the reperfusion phase rapidly converts that susceptibility into an ROS burst and downstream injury cascades (Figure 1). Accordingly, the severity of MIRI depends not only on the balance between ROS generation and clearance after reoxygenation but also on the pre-reoxygenation state of metabolic substrates, respiratory-chain function, and mitochondrial quality (43, 44).

**Figure 1:**
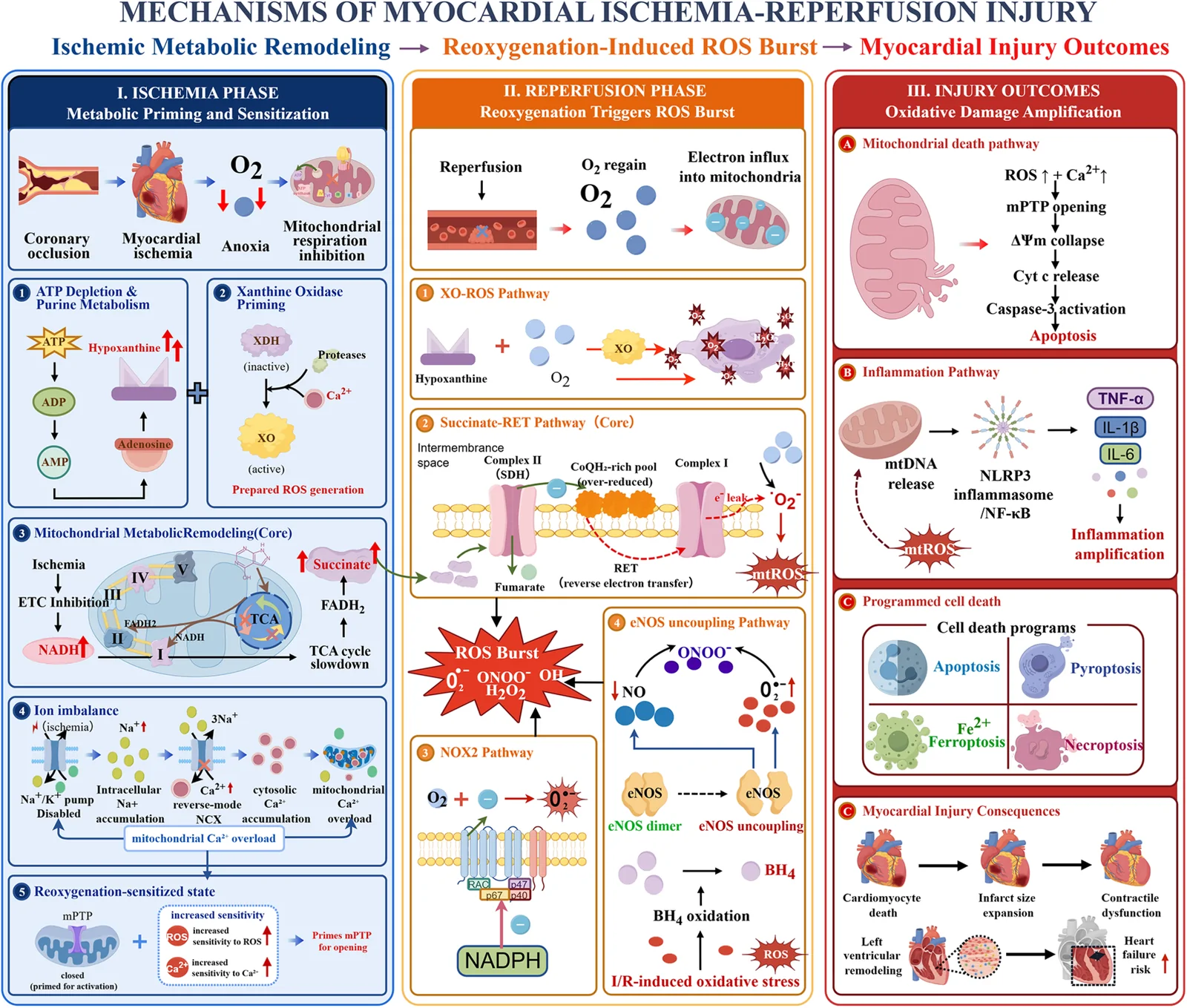
The comprehensive mechanism of myocardial ischemia-reperfusion injury. **Ⅰ**. During the ischemic stage, ATP depletion, xanthine oxidase activation, mitochondrial metabolic remodeling, ion imbalance, and reperfusion sensitization all contribute to increasing the susceptibility to oxidative damage. **Ⅱ.** During reperfusion, rapid reoxygenation triggers the release of reactive oxygen species through multiple pathways, including xanthine oxidase, reverse electron transfer (succinate-RET), NOX2 and eNOS uncoupling, and BH4 oxidation. **Ⅲ.** The resulting oxidative stress activates mitochondrial death pathways, exacerbates inflammatory responses, and triggers various programmed cell deaths, ultimately leading to adverse myocardial injury outcomes.

### 3.2 Temporal limitations of endogenous defense

The endogenous myocardial protective system comprises constitutive antioxidant enzymes, stress-inducible transcriptional programs, and mechanisms involved in damage repair and organelle quality control (45). Superoxide dismutase (SOD), catalase (CAT), and glutathione peroxidase (GPx) can directly remove superoxide, hydrogen peroxide, and lipid hydroperoxides; however, their basal reserves and response capacity are limited and can rapidly approach saturation under the high-flux ROS production of early reperfusion (46, 47). Inducible defenses typified by the Nrf2-antioxidant response element (ARE) pathway can enhance glutathione synthesis, peroxide reduction, quinone detoxification, and protection against lipid peroxidation, but Nrf2 stabilization and nuclear translocation must be followed by target-gene transcription, translation, and accumulation of effector proteins, all of which require time (12, 13). By contrast, the ROS burst, membrane lipid peroxidation, mPTP opening, and respiratory-chain injury can occur rapidly during early reperfusion, creating a temporal mismatch between the initiation of injury and the establishment of a fully effective defense (48–50).

HIF-1*α*-mediated hypoxic adaptation is subject to a similar temporal limitation (51). The fall in oxygen tension during ischemia promotes HIF-1*α* stabilization and progressively initiates programs that regulate glycolytic energy production, mitochondrial substrate utilization, and organelle quality. However, because this response is triggered by pathological hypoxia itself, its functional output still requires protein stabilization, transcriptional activation, and development of downstream effects. Meanwhile, ATP depletion, acidosis, ionic disequilibrium, and mitochondrial dysfunction progress in parallel with ischemia (52, 53). Consequently, when HIF-1*α* and Nrf2 are activated primarily by endogenous stress signals arising during ischemia or after reperfusion, their protective effects may lag behind key pathological events.

This kinetic mismatch makes the timing of intervention a major determinant of cardioprotection. ROS scavenging after reperfusion is constrained by tissue delivery, subcellular localization, and the spectrum of reactive species targeted, and it cannot readily correct the metabolic abnormalities and mitochondrial damage already established during ischemia. Preconditioning applies brief, controlled stimuli before severe ischemia and reperfusion, allowing adaptive programs to be initiated while myocardial homeostasis remains relatively intact and thereby shortening the temporal gap between injury onset and protective effector formation (54, 55). Its value is therefore not limited to advancing the antioxidant response; rather, it shifts the overall protective window to a period before ischemia-reperfusion injury is fully established.

Nevertheless, simply increasing ROS-scavenging capacity during reperfusion is insufficient to address the continuous pathological course of MIRI. The magnitude of the oxidative burst after reoxygenation is determined to a large extent by the metabolic substrate profile, respiratory-chain function, and mitochondrial quality established during ischemia, whereas subsequent expansion of injury is constrained by myocardial redox-defense capacity (56–58). An effective preconditioned state should therefore establish both metabolic adaptation and antioxidant defense: the former reduces oxidative susceptibility by improving energy metabolism and mitochondrial status before reoxygenation, whereas the latter increases tolerance to the oxidative burden of reperfusion by enhancing glutathione metabolism, peroxide clearance, and protection against lipid peroxidation. HIF-1*α* and Nrf2 occupy key positions in these two protective programs, respectively. Whether they form directional regulatory links and can be recruited in an ordered manner by preconditioning is therefore central to the mechanistic analysis that follows.

## 4 Endogenous adaptation and defense

### 4.1 HIF-1*α*-mediated hypoxic adaptation

Hypoxia-inducible factors (HIFs) are central transcriptional regulators of cellular adaptation to hypoxia (59, 60). Both HIF-1*α* and HIF-2*α* heterodimerize with constitutively expressed HIF-1β, also known as aryl hydrocarbon receptor nuclear translocator (ARNT), and recognize hypoxia response elements (HREs); however, the two isoforms differ in response kinetics and functional emphasis (61). HIF-1*α* primarily responds to acute hypoxia and rapidly regulates glycolysis, mitochondrial oxygen consumption, and cell survival, whereas HIF-2*α* is more strongly associated with angiogenesis, erythropoiesis, and tissue repair during chronic hypoxia (60, 62). Because the critical pathological changes in MIRI occur during the acute transition from ischemia to reoxygenation over minutes to hours, HIF-1*α* can regulate energy metabolism and mitochondrial function on the relevant timescale and thus represents an important node linking metabolic adaptation during ischemia to oxidative injury during reperfusion (56).

HIF-1*α* protein homeostasis is governed primarily by the prolyl hydroxylase domain protein (PHD)-von Hippel-Lindau protein (VHL) oxygen-sensing system (63). Under normoxic conditions, *HIF1A* maintains basal transcription and protein synthesis, while PHDs use O2, Fe2+, and *α*-ketoglutarate as cofactors to hydroxylate Pro402 and Pro564 within the oxygen-dependent degradation domain of HIF-1*α* (64). Hydroxylated HIF-1*α* is recognized by VHL and rapidly degraded through the ubiquitin-proteasome pathway, maintaining low intracellular abundance (61, 64). When ischemia lowers oxygen tension, PHD catalytic activity is inhibited, allowing newly synthesized HIF-1*α* to escape VHL-mediated degradation because of insufficient hydroxylation and to accumulate progressively. HIF-1*α* then translocates to the nucleus, heterodimerizes with HIF-1β, and binds HREs within regulatory regions of target genes (59, 61, 63, 64). Factor inhibiting HIF (FIH) can hydroxylate Asn803 in the C-terminal transactivation domain of HIF-1*α*, thereby limiting interaction with coactivators such as p300/CBP and further constraining HIF-1*α* transcriptional output (65–67).

Beyond the canonical oxygen-sensing mechanism, metabolic, inflammatory, and cell-survival signals within the MIRI microenvironment also regulate HIF-1*α* homeostasis (68, 69). Redox fluctuations during ischemia-reoxygenation can alter the availability or redox state of Fe2 + required for PHD catalysis, thereby affecting HIF-1*α* hydroxylation and degradation (59, 69). Inflammatory mediators such as tumor necrosis factor-α (TNF-α) and interleukin-1β (IL-1β) can increase *HIF1A* transcription through nuclear factor-*κ*B (NF-*κ*B) (74, 75), whereas phosphatidylinositol 3-kinase/protein kinase B/mechanistic target of rapamycin (PI3 K/Akt/mTOR) and mitogen-activated protein kinase/extracellular signal-regulated kinase (MAPK/ERK) signaling can promote HIF-1*α* translation or enhance its nuclear transcriptional activity, respectively (68, 69). In addition, ischemia-associated metabolic disturbances can increase tricarboxylic acid cycle intermediates such as succinate and fumarate, which under certain conditions competitively inhibit *α*-ketoglutarate-dependent dioxygenases, including PHDs, thereby reducing HIF-1*α* hydroxylation and promoting its stabilization (70, 71). HIF-1*α* is therefore not a simple hypoxia-responsive switch but a dynamic regulatory node that integrates oxygen tension, metabolism, and multiple stress signals.

Once stabilized and transcriptionally active, HIF-1*α* can increase myocardial tolerance to ischemia and reperfusion through multiple levels of regulation (72). It first induces glycolysis-related molecules, including GLUT1, HK2, PFKFB3, and LDHA, thereby increasing glucose uptake and glycolytic ATP production and allowing the myocardium to maintain a degree of ATP generation when oxidative phosphorylation is restricted (73, 74). HIF-1*α* also upregulates PDK1 and suppresses PDH activity, reducing pyruvate entry into the tricarboxylic acid cycle and shifting glucose metabolism from mitochondrial oxidation toward cytosolic glycolysis, thereby reducing the oxygen dependence of energy production (75, 76). Reduced mitochondrial substrate input through PDK1, together with regulation of respiratory-chain composition and activity by NDUFA4L2 and COX4 isoform switching, can further decrease mitochondrial oxygen consumption and electron-transfer pressure during hypoxia, limit respiratory-chain over-reduction and the formation of damaged mitochondria, and thereby weaken the metabolic and organelle basis for the ROS burst after reoxygenation (77–79). HIF-1*α* also induces BNIP3 and BNIP3L/NIX to participate in mitochondrial quality control, promoting removal of dysfunctional mitochondria and limiting the pre-reoxygenation accumulation of mitochondria with depolarized membranes, impaired respiratory chains, and a propensity for ROS generation (80–82). In addition, HIF-1*α* can reduce energy expenditure and inflammatory activation through ecto-5′-nucleotidase (CD73)-adenosine signaling (83), improve microcirculatory adaptation in ischemic tissue by inducing vascular endothelial growth factor (VEGF) and related vascular responses, and participate in the regulation of iron uptake, storage, and export, thereby influencing the labile iron pool and susceptibility to lipid peroxidation (84–86). Overall, the cardioprotective role of HIF-1*α* extends beyond maintenance of energy supply during hypoxia; it also remodels the metabolic and mitochondrial state before reoxygenation, reducing the substrate for severe oxidative stress after reperfusion.

The biological effects of HIF-1*α* are strongly dependent on timing and magnitude (87, 88). During sustained severe ischemia, PHDs and FIH remain inhibited because of profound oxygen deprivation. Although HIF-1*α* remains stabilized, the myocardium concurrently develops ATP depletion, acidosis, ionic imbalance, and mitochondrial damage, which constrain its adaptive output (88, 89). Prolonged maintenance of a glycolytic shift, PDH inhibition, or BNIP3-related mitochondrial regulation may increase the risk of impaired metabolic recovery and disrupted organelle homeostasis (90–92). By contrast, preconditioning occurs before severe injury, when myocardial energy reserves, mitochondrial structure, and oxygen-sensing regulatory systems remain relatively intact. Brief stimulation can induce moderate HIF-1*α* stabilization and establish downstream protective programs in advance; once the stimulus is withdrawn and oxygen supply is restored, PHD-VHL-mediated degradation and FIH-mediated restriction of transcriptional activity can be re-established, conferring substantial reversibility and endogenous restraint on HIF-1*α* activation (89).

Overall, HIF-1*α* functions both as an effector of metabolic and mitochondrial adaptation during ischemia and as a regulatory node integrating redox, inflammatory, and cell-survival signals. This multilayered regulation provides a molecular basis for directional interactions between HIF-1*α* and other stress-response pathways.

### 4.2 Nrf2-mediated antioxidant defense

Nrf2 is a central transcriptional regulator of inducible cellular redox defense. Unlike constitutive antioxidant enzymes that directly scavenge ROS, Nrf2 expands antioxidant capacity programmatically by coordinating the expression of genes involved in reducing-substrate synthesis, peroxide detoxification, quinone detoxification, and membrane-lipid protection (93–95). Its principal role is therefore not to neutralize a single ROS species immediately, but to raise the overall threshold at which the myocardium can tolerate the acute oxidative burden of reperfusion.

Under basal conditions, Kelch-like ECH-associated protein 1 (Keap1) acts as the substrate adaptor for the Cullin 3 (Cul3)/RING-box protein 1 (Rbx1) E3 ubiquitin ligase complex and continuously promotes Nrf2 ubiquitination and proteasomal degradation (93–95). Oxidative or electrophilic stress modifies reactive cysteine residues in Keap1, altering its conformation and ubiquitination function and allowing newly synthesized Nrf2 to escape degradation and accumulate in the cytoplasm (96). Stabilized Nrf2 translocates to the nucleus, heterodimerizes with small Maf proteins (sMaf), binds AREs in regulatory regions of target genes, and initiates cytoprotective transcriptional programs (97, 98). In addition to the canonical Keap1-sensing mechanism, competitive binding of p62/sequestosome 1 (p62/SQSTM1) to Keap1, PI3 K/Akt-mediated inhibition of the glycogen synthase kinase 3*β* (GSK3*β*)-β-transducin repeat-containing protein (*β*-TrCP) degradation pathway, and AMP-activated protein kinase (AMPK)-mediated phosphorylation and nuclear translocation of Nrf2 can all modulate its stability and transcriptional activity (99–102). These noncanonical pathways allow signals associated with energetic stress, mitochondrial injury, and preconditioning to activate Nrf2 before a high-intensity ROS burst, thereby establishing antioxidant defense in advance of reperfusion.

The protective transcriptional output of Nrf2 can engage redox-defense programs at multiple levels. Activated Nrf2 translocates to the nucleus and binds AREs, upregulating a broad set of downstream target genes. The catalytic and modifier subunits of glutamate-cysteine ligase (GCLC/GCLM) and solute carrier family 7 member 11 (SLC7A11) increase cystine uptake and promote glutathione (GSH) synthesis. As the major intracellular low-molecular-weight reducing buffer, GSH is an essential substrate for the detoxifying activity of GPx and glutathione peroxidase 4 (GPX4), while GPx and GPX4 facilitate the removal of peroxides and membrane lipid hydroperoxides (103–105). NAD(P)H:quinone oxidoreductase 1 (NQO1) limits quinone redox cycling and secondary ROS generation, whereas heme oxygenase 1 (HO-1) catalyzes heme degradation to biliverdin, CO, and Fe2 + and contributes to heme clearance and adaptation to oxidative stress (106–110). Collectively, these effects help preserve mitochondrial membrane lipids, respiratory-chain proteins, and ion-transport proteins and restrict iron-dependent lipid peroxidation through the SLC7A11-GSH-GPX4 axis and regulation of iron metabolism (111). Nrf2 therefore limits progression from reperfusion-associated oxidative stress to mitochondrial failure and irreversible cell injury by strengthening reducing capacity, peroxide disposal, and membrane-lipid protection.

Nrf2-mediated protection is likewise strongly time dependent. If Nrf2 activation relies primarily on oxidative modification of Keap1 by reperfusion-derived ROS, sufficient time is still required for protein stabilization, nuclear translocation, ARE binding, and accumulation of downstream protective proteins, whereas key injuries such as the mitochondrial ROS burst, membrane lipid peroxidation, and mPTP opening can occur rapidly during early reperfusion. The value of preconditioning lies in increasing Nrf2 activity before severe oxidative stress through Keap1-dependent mechanisms or noncanonical pathways such as PI3 K/Akt-GSK3*β* and AMPK, allowing effector proteins involved in GSH metabolism, peroxide clearance, and defense against lipid peroxidation to accumulate in advance (112–117). In this way, Nrf2-mediated defense can be transformed from a damage-induced response into a functional reserve already present before reperfusion, better matching the temporal characteristics of early oxidative injury in MIRI.

Taken together, HIF-1*α*-mediated hypoxic/metabolic adaptation and Nrf2-mediated antioxidant defense are functionally complementary in MIRI **(**Figure 2**).** Whether this complementarity reflects directional regulatory coupling, rather than parallel activation by shared stress signals, requires causal evidence in both directions.

**Figure 2:**
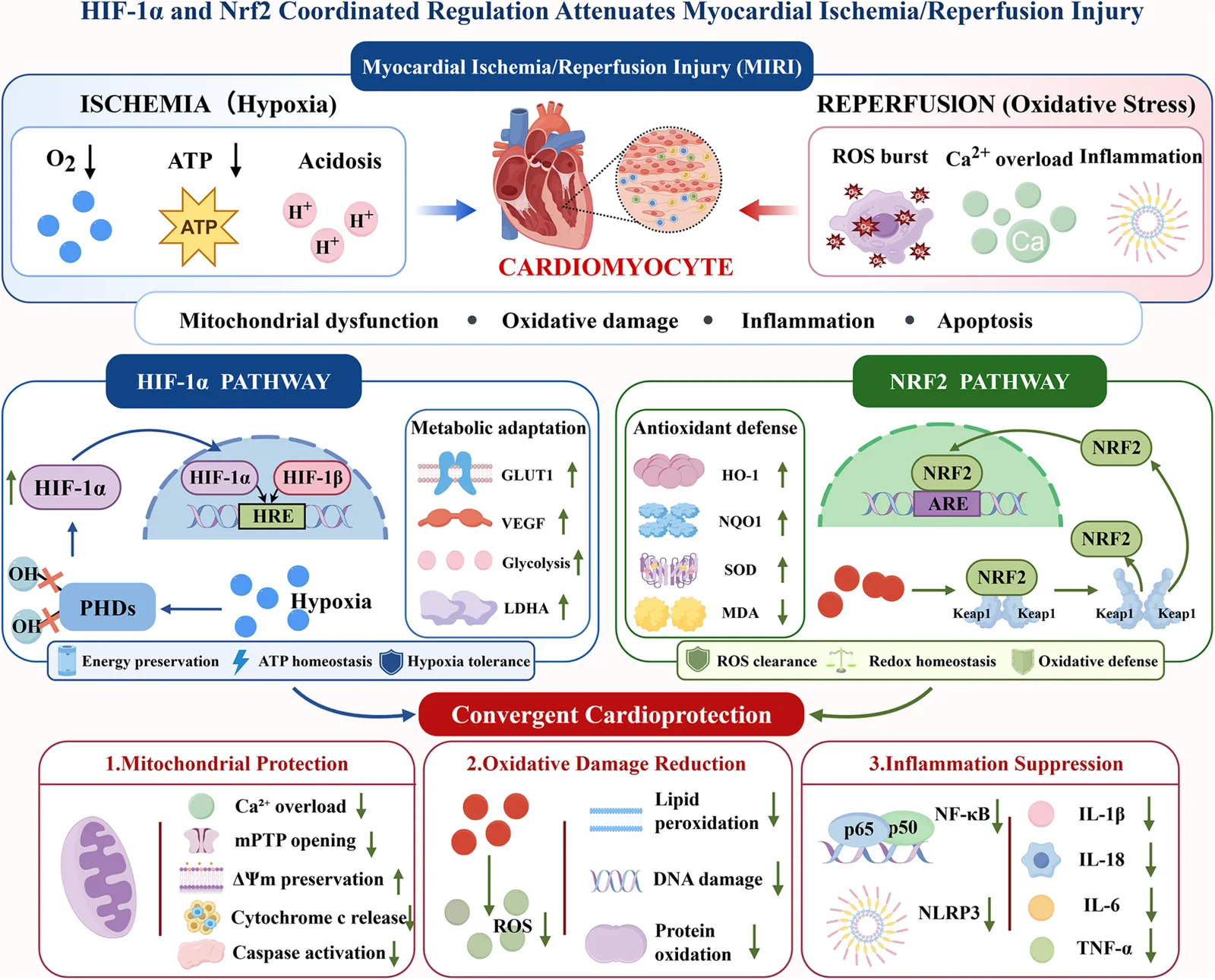
Convergent cardioprotective mechanisms mediated by the HIF-1*α* and Nrf2 pathways during myocardial ischemia–reperfusion injury. Ischemia-induced hypoxia is characterized by reduced oxygen availability, ATP depletion, and acidosis, whereas reperfusion triggers excessive ROS generation, Ca^2^⁺ overload, and inflammation. Under hypoxic conditions, stabilization of HIF-1*α* promotes transcription of hypoxia-responsive genes involved in metabolic adaptation, glycolysis, angiogenesis, and cellular survival. In parallel, activation of Nrf2 induces antioxidant and cytoprotective genes through antioxidant response elements (AREs), enhancing ROS clearance and redox homeostasis. The coordinated actions and crosstalk of HIF-1*α* and Nrf2 converge to protect mitochondria, reduce oxidative damage, and suppress inflammatory signaling, thereby limiting myocardial ischemia–reperfusion injury.

## 5 The HIF-1*α*-Nrf2 regulatory network

### 5.1 Functional significance

In the context of the temporal pathophysiology of MIRI, the principal significance of bidirectional HIF-1*α*–Nrf2 regulation is its potential to integrate metabolic adaptation during ischemia with redox defense during reperfusion into a continuous endogenous protective system. Following HIF-1*α* stabilization induced by hypoxia or preconditioning stimuli, HIF-1*α* not only limits metabolic disturbances and organelle susceptibility to oxidative injury before reoxygenation through metabolic reprogramming, reduced mitochondrial oxygen consumption, and enhanced mitochondrial quality control, but also promotes Nrf2 gene expression, protein accumulation, and transcriptional activation, thereby initiating antioxidant defense programs before the onset of reperfusion. Hypoxic adaptation signals initiated during ischemia may therefore be extended into redox defense during reperfusion, shortening the temporal gap between the ROS burst and the emergence of antioxidant protection.

Reciprocal support of HIF-1*α* by Nrf2 may further preserve continuity of this protective state across the ischemia–reoxygenation transition. In addition to enhancing GSH metabolism, facilitating ROS clearance, and counteracting lipid peroxidation, Nrf2 can regulate HIF-1*α* at multiple levels—*HIF1A* transcription, post-transcriptional regulation, and protein stability—thereby sustaining HIF-1*α*-mediated adaptive responses. During early reperfusion, when mitochondrial oxidative phosphorylation has not yet been fully restored, reoxygenation promotes rapid PHD-mediated degradation of HIF-1*α*; Nrf2-mediated support at this stage may help preserve compensatory glycolytic ATP production, support ion-pump function, and facilitate the restoration of calcium homeostasis, providing a metabolic buffer during the return from hypoxic metabolism to mitochondrial oxidative energy production. The two directions of support thus both lower oxidative susceptibility before reoxygenation and raise oxidative tolerance during reperfusion, maintaining protection across the ischemia–reoxygenation transition.

The therapeutic value of this coupling therefore lies not in continuously amplifying either node, but in recruiting the network in the appropriate sequence and for the appropriate duration. Whereas pathological ischemia or post-reperfusion injury activates the pathways reactively and often too late, preconditioning applies brief, controlled stimuli before injury, while myocardial homeostasis and negative-feedback restraint remain intact, engaging the network moderately and reversibly rather than sustaining it. Preconditioning thus provides a practical entry point for translating the HIF-1*α*–Nrf2 network into cardioprotection in MIRI.

### 5.2 Regulation of Nrf2 by HIF-1*α*

Both HIF-1*α* and Nrf2 respond to hypoxia, redox changes, and preconditioning stimuli; therefore, their simultaneous elevation does not in itself demonstrate a regulatory relationship. More mechanistically informative evidence is provided when changes in HIF-1*α* function are followed by corresponding changes in Nrf2 expression, protein stability, nuclear translocation, or ARE-dependent transcriptional activity. Current studies suggest that HIF-1*α* can transmit hypoxia-sensing signals into antioxidant defense programs by promoting Nrf2 expression and enhancing its functional activation.

In studies related to myocardial ischemic preconditioning, Jiang et al. reported that dihydrotanshinone I transiently inhibits mitochondrial respiratory-chain complex I, inducing moderate mitochondrial ROS production and stabilizing HIF-1*α* (118). HIF-1*α* subsequently increased Nrf2 promoter activity, transcription, protein expression, and nuclear accumulation, together with HO-1 and NQO1 expression, whereas HIF-1*α* knockdown markedly attenuated these effects **(**Figure 3A**)**. These findings support HIF-1*α*-dependent transcriptional regulation and functional activation of Nrf2; however, direct binding of HIF-1*α* to the NFE2L2 promoter has not been demonstrated, leaving the underlying transcriptional mechanism unresolved. In hypoxia models, HIF-1*α* deficiency reduced Nrf2 protein accumulation, impaired nuclear translocation, and decreased Nrf2-ARE binding, whereas enhanced HIF-1*α* promoted Nrf2 activation and downstream antioxidant-gene expression, further supporting a role for HIF-1*α* in regulating the functional state of Nrf2 (15).

**Figure 3:**
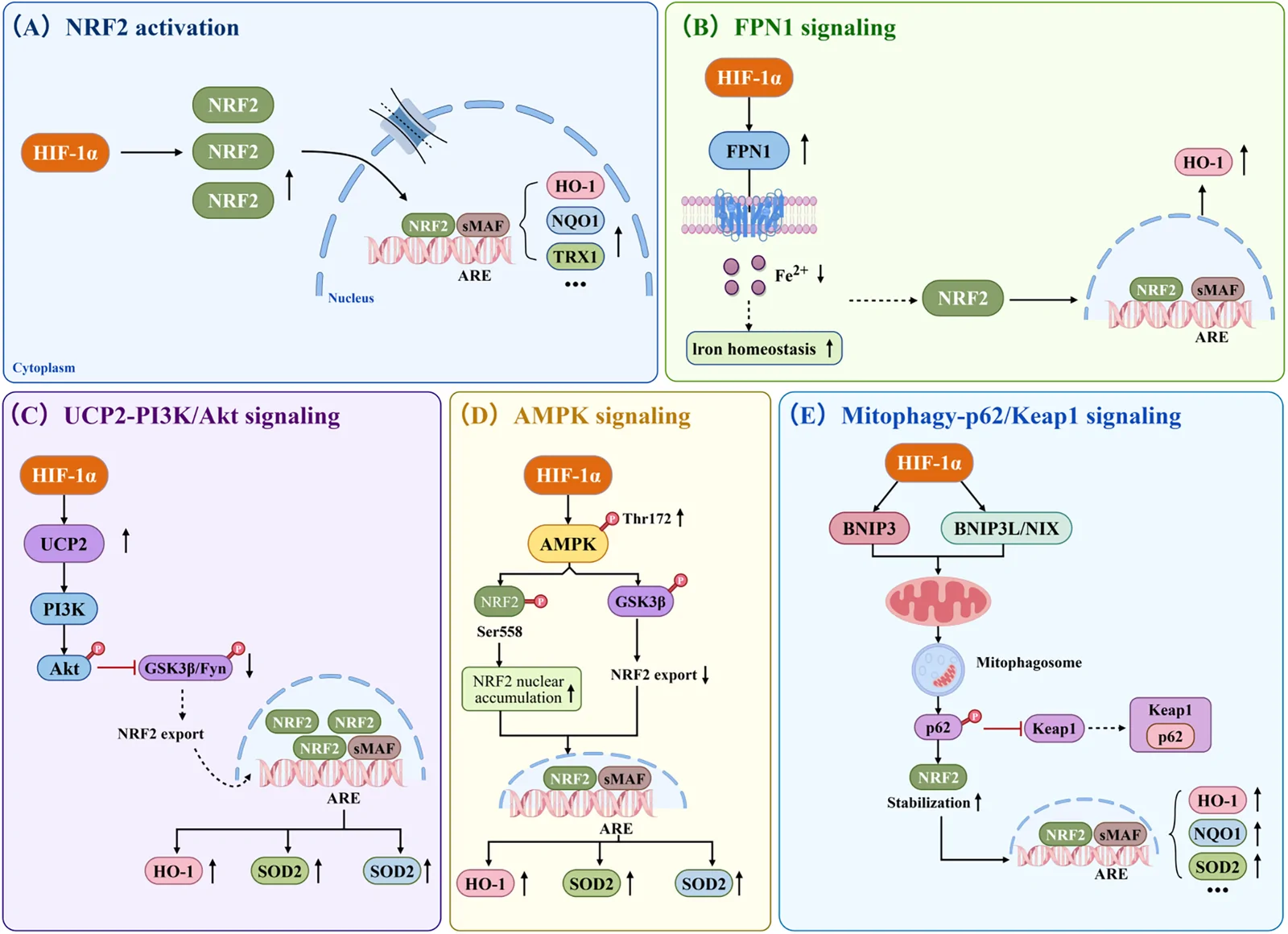
Proposed mechanisms by which HIF-1*α* promotes Nrf2 activation. **(A)** HIF-1*α* promotes Nrf2 expression and functional activation, increasing Nrf2 nuclear accumulation and ARE-dependent antioxidant transcription. **(B)** HIF-1*α*-induced FPN1 expression may improve iron homeostasis and facilitate Nrf2/HO-1 signaling. **(C)** HIF-1*α*-induced UCP2 activates PI3K/Akt signaling, which suppresses GSK3*β*/Fyn-dependent Nrf2 nuclear export and sustains ARE-dependent transcription. **(D)** HIF-1*α*-associated AMPK activation promotes Nrf2 phosphorylation and nuclear accumulation while limiting GSK3*β*-mediated Nrf2 export. **(E)** HIF-1*α*-induced BNIP3/BNIP3L/NIX-mediated mitophagy may activate the p62/Keap1/Nrf2 axis, thereby stabilizing Nrf2 and enhancing antioxidant-gene expression.

Beyond direct regulation, HIF-1*α* may modulate Nrf2 activity through metabolic and signaling intermediates. In models of cerebral ischemia-reperfusion and oxygen-glucose deprivation/reoxygenation, HIF-1*α* activation increased ferroportin 1 (FPN1) expression and enhanced Nrf2/HO-1 signaling, whereas FPN1 inhibition weakened the protective effect (119). Because FPN1 regulates cellular iron homeostasis and oxidative injury, it may serve as a metabolic relay linking HIF-1*α*-mediated hypoxic adaptation to the Nrf2 antioxidant response (Figure 3B). In addition, stabilization of HIF-1*α* in models of diabetic myocardial injury upregulated mitochondrial uncoupling protein 2 (UCP2), which subsequently activated PI3 K/Akt signaling and increased Nrf2 and its downstream antioxidant molecules HO-1 and SOD2; loss of UCP2 markedly attenuated these antioxidant effects. Mechanistically, myocardial I/R studies have shown that Akt can inhibit the GSK3*β*/Fyn axis, reduce Nrf2 nuclear export, and prolong its nuclear retention, thereby sustaining ARE-dependent antioxidant transcription. These findings support PI3 K/Akt as a potential interface between HIF-1*α*-related adaptation and Nrf2 activation (Figure 3C) (120–122).

AMPK may provide an additional metabolic signaling node linking HIF-1*α*-mediated hypoxic adaptation to Nrf2-dependent antioxidant defense. Hypoxia-induced stabilization of HIF-1*α* is accompanied by AMPK activation, including phosphorylation of AMPK at Thr172 and remodeling of cellular energy metabolism (123). Activated AMPK can phosphorylate Nrf2 at Ser558, promote its nuclear accumulation, and enhance ARE-dependent transcription; AMPK can also further reduce Nrf2 nuclear export by inhibiting GSK3*β* (124). Thus, HIF-1*α*-AMPK-Nrf2 signaling may connect hypoxic adaptation and energy-metabolic regulation to Nrf2-mediated redox defense (Figure 3D). Mitochondrial quality control may provide another indirect link. Hypoxia-stabilized HIF-1*α* induces BNIP3 and BNIP3L/NIX, promoting receptor-mediated mitophagy and selective removal of damaged mitochondria (125). In hypoxia/ischemia-related models, adaptive BNIP3-mediated mitophagy has been associated with activation of the p62/SQSTM1-Keap1-Nrf2 axis, promoting Nrf2 stabilization, nuclear translocation, and ARE-dependent antioxidant transcription (126, 127). Because BNIP3/NIX can also promote mitochondrial dysfunction and cell-death signaling when excessively or persistently activated (91, 125), this protective link is likely dependent on the magnitude and duration of the mitophagic response. Thus, HIF-1*α* may indirectly strengthen Nrf2 defense through BNIP3/NIX-mediated mitochondrial quality control (Figure 3E).

Collectively, regulation of Nrf2 by HIF-1*α* may occur at multiple levels, including transcription, protein homeostasis, and signaling relay. HIF-1*α* may promote Nrf2 transcription, enhance Nrf2 stability and nuclear translocation, and strengthen Nrf2-dependent antioxidant transcription through intermediates such as FPN1 and UCP2-PI3 K/Akt. In parallel, mechanisms involving AMPK and BNIP3/NIX-mitophagy-p62/Keap1 further suggest that energetic stress and mitochondrial quality control may constitute functional interfaces between HIF-1*α* and Nrf2. Together, these multiple layers of direct and indirect mechanisms provide a molecular basis for extending HIF-1*α*-mediated hypoxic adaptation into Nrf2-mediated redox defense.

### 5.3 Regulation of HIF-1*α* by Nrf2

Direct transcriptional regulation of *HIF1A* represents one of the clearest mechanisms by which Nrf2 can influence HIF-1*α* signaling. In breast cancer cells, a highly conserved ARE located approximately 32 kb upstream of the *HIF1A* transcription start site is occupied and activated by the Nrf2–sMaf complex, as demonstrated by chromatin immunoprecipitation, protein–DNA binding, and reporter assays (128). Mutation of this ARE using clustered regularly interspaced short palindromic repeats (CRISPR)/Cas9 genome editing reduced Nrf2 occupancy, basal HIF1A mRNA and HIF-1*α* protein levels, and downstream HIF target-gene expression, supporting HIF1A as a noncanonical direct transcriptional target of Nrf2 **(**Figure 4A**)** (128). Notably, this regulatory effect is detectable under basal, nonhypoxic conditions, indicating that Nrf2 can actively contribute to *HIF1A* transcription independently of acute hypoxic stimulation. This transcriptional input increases the availability of *HIF1A* for subsequent translation but does not bypass oxygen-dependent control of HIF-1*α* stability by the PHD–VHL system, thereby providing a larger transcriptional substrate for HIF-1*α* accumulation when oxygen tension falls.

**Figure 4:**
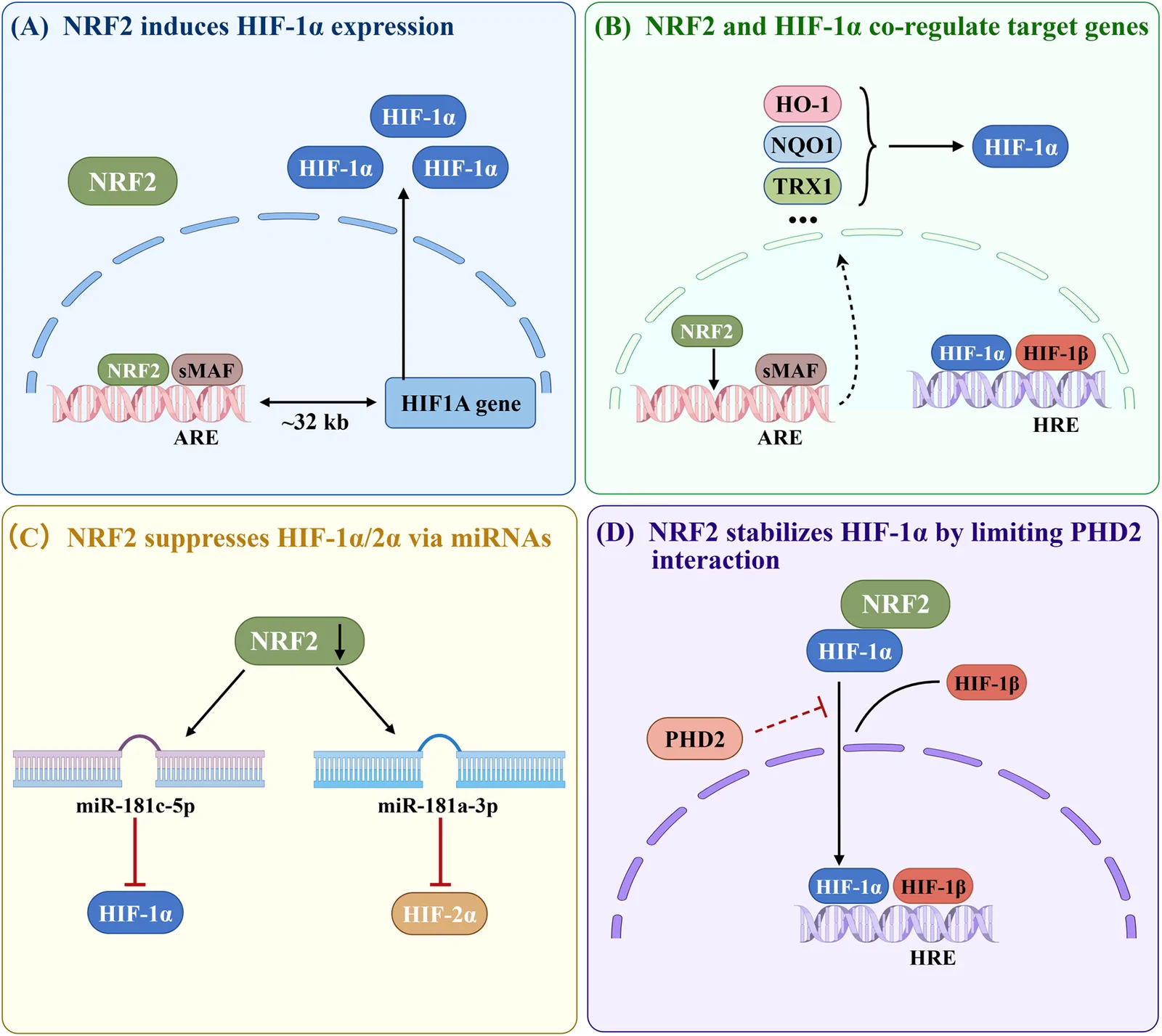
Representative mechanisms underlying the regulation of HIF signaling by Nrf2. **(A)** Nrf2 directly promotes HIF1A transcription by binding to a conserved antioxidant response element (ARE) located approximately 30 kb upstream of the HIF1A gene. **(B)** Nrf2-dependent target genes, including HO-1, NQO1, and TRX1, can enhance HIF-1*α* accumulation and activity, thereby linking antioxidant defense to hypoxia-responsive signaling. **(C)** Nrf2 regulates HIF-1*α* and HIF-2*α* through microRNA-dependent mechanisms; loss or inhibition of Nrf2 increases miR-181c-5p and miR-181a-3p, which suppress HIF-1*α* and HIF-2*α*, respectively. **(D)** Nrf2 can directly interact with the oxygen-dependent degradation domain of HIF-1*α* and interfere with its association with PHD2, thereby reducing HIF-1*α* hydroxylation and proteasomal degradation.

In breast cancer cells, in addition to direct transcriptional regulation, Nrf2 may sustain HIF-1*α* accumulation through a post-transcriptional mechanism involving microRNA-181c (miR-181c) (129). Nrf2 silencing increases miR-181c and is accompanied by reduced hypoxia-induced HIF-1*α* and lower expression of metabolic target genes such as GLUT1, HK2, PDK1, and LDHA. Overexpression of miR-181c reproduces this inhibitory phenotype, whereas inhibition of miR-181c in the context of Nrf2 silencing partially restores HIF-1*α* accumulation and its glycolytic transcriptional program. These findings identify miR-181c as a functional post-transcriptional relay between Nrf2 and HIF-1*α* **(**Figure 4C**)**.

Nrf2 may further influence HIF-1*α* at the level of protein stability. In hepatocellular carcinoma models, Nrf2 has been shown to physically interact with the oxygen-dependent degradation domain of HIF-1*α*, limiting PHD2-mediated hydroxylation and subsequent VHL-dependent ubiquitination and proteasomal degradation, thereby increasing HIF-1*α* accumulation and transcriptional activity **(**Figure 4D**)** (130). Similarly, the Nrf2 target protein NQO1 can bind the oxygen-dependent degradation region of HIF-1*α* and compete with PHDs, further prolonging HIF-1*α* protein stability (131). These findings indicate that Nrf2 signaling can potentially reinforce HIF-1*α* not only by increasing its transcriptional supply but also by limiting its post-translational degradation. In arsenic-exposed bronchial epithelial cells, Nrf2 inhibition or deletion reduced HIF-1*α* induction (132). In renal tubular epithelial cells, Nrf2 maintained basal HIF1A transcription and supported hypoxia-induced HIF-1*α* accumulation (133). Shared regulation of protein turnover can also produce divergent responses: in HEK293 cells, UBXN7 deficiency increased Nrf2 abundance while reducing HIF-1*α* levels (134). Nrf2-dependent downstream effectors may provide an additional route for sustaining HIF-1*α* activity. Under intermittent hypoxia, moderate ROS signaling mediated by NADPH oxidase 1 (NOX1) can sequentially activate Nrf2 and its target thioredoxin 1 (Trx1), which in turn enhances HIF-1*α* accumulation and HIF-dependent transcription (135). Notably, continuous hypoxia in the same study induced HIF-1*α* without comparable activation of the Nrf2/Trx1 pathway, indicating that the NOX1–Nrf2–Trx1 branch primarily functions as a redox-sensitive amplification mechanism superimposed on oxygen-dependent HIF-1*α* regulation rather than as the primary oxygen-sensing pathway. Together with NQO1-mediated HIF-1*α* stabilization, these findings show how Nrf2-dependent effectors can reinforce HIF signaling under specific redox conditions **(**Figure 4B**)**.

Although several of these mechanisms were defined in non-cardiac experimental systems, they involve relatively proximal molecular events, including a conserved cis-regulatory ARE, sequence-dependent microRNA regulation, direct protein interactions with the HIF-1*α* degradation machinery, and redox-sensitive regulation of HIF-1*α* stability. Because these mechanisms act directly at the levels of gene regulation, protein turnover, and redox control, they are less dependent on tissue-specific signaling architecture than more distal phenotypic responses and therefore provide a reasonable mechanistic basis for extrapolation to MIRI. This relevance is further supported by the fact that myocardial ischemia–reperfusion engages the same PHD–VHL/HIF oxygen-sensing system and Nrf2-centered redox-response machinery. Accordingly, these non-cardiac studies provide mechanistic support for potential Nrf2-to-HIF-1*α* regulation in the ischemic heart, while the relative contribution of individual regulatory routes is likely to vary with the cardiac and ischemia–reperfusion context.

Overall, Nrf2 may support HIF-1*α* signaling through complementary regulatory layers, including direct enhancement of *HIF1A* transcription, miR-181c-mediated post-transcriptional regulation, protection of HIF-1*α* from PHD–VHL-dependent degradation, and induction of downstream effectors such as NQO1 and Trx1 that further sustain HIF-1*α* stability and activity. These mechanisms provide a molecular framework through which Nrf2-dependent redox defense may reinforce hypoxic and metabolic adaptation during ischemia and early reoxygenation. Together with HIF-1*α*-dependent recruitment of Nrf2 described above, the available evidence supports a potentially reciprocal coupling between hypoxic adaptation and antioxidant defense at the levels of transcription, protein homeostasis, and redox signaling. Representative experimental evidence supporting these regulatory mechanisms is summarized in Table 1.

**Table 1:** Representative experimental evidence for bidirectional HIF-1α–Nrf2 regulation and relevance to MIRI.

Reference	Study model	Regulatory intervention	Changes in regulated node	Molecular mechanism	MIRI relevance / evidentiary role
Evidence for HIF-1α → Nrf2 regulation					
(15)	Mouse skeletal muscle and C2C12 myoblasts; acute hypoxia	HIF-1α siRNA/genetic enhancement; graded acute hypoxia	HIF-1α loss reduced Nrf2 accumulation, nuclear translocation, ARE binding, and target-gene expression; HIF-1α enhancement increased the Nrf2–ARE response	HIF-1α → Nrf2 functional activation → ARE-dependent transcription	Hypoxia-context causal evidence; supports HIF-1α→Nrf2 activation relevant to ischemic adaptation, but not myocardial I/R.
(118)	Myocardial I/R-related pharmacological preconditioning; myocardium/cardiomyocytes	Dihydrotanshinone I; HIF-1α KD/inhibition; sequential protocatechualdehyde	HIF-1α stabilization increased Nrf2 promoter activity, expression, nuclear accumulation, and HO-1/NQO1; HIF-1α inhibition attenuated these effects	Complex I inhibition → moderate ROS → HIF-1α stabilization → Nrf2 transcription/ARE output	Direct MIRI-context evidence; supports HIF-1α-dependent Nrf2 recruitment during myocardial I/R-related preconditioning.
(119)	Cerebral I/R and neuronal/PC12 OGD/R models	HIF-1α activation/OE; FPN1 inhibition/KD	HIF-1α activation increased FPN1 and Nrf2/HO-1 signaling; FPN1 blockade weakened the protective response	HIF-1α → FPN1 → Nrf2/HO-1	I/R-context support; non-cardiac I/R/OGD/R validates an FPN1 relay with potential transferability to MIRI.
(120–122)	Diabetic myocardial injury/high-glucose-related models; myocardium and cardiomyocytes	Dihydrotanshinone I, roxadustat, or sulforaphane; HIF-1α/UCP2 or PI3K–Akt pathway modulation	HIF-1α/UCP2 signaling and Akt activation were associated with increased Nrf2 output; HIF-1α/UCP2 loss weakened antioxidant responses	HIF-1α → UCP2 → PI3K/Akt ⊣ GSK3β/Fyn → Nrf2 nuclear retention	Cardiac-context support; links HIF-1α/UCP2–PI3K/Akt to Nrf2, but not yet as a complete MIRI cascade.
(136)	Aged-rat RIPC followed by myocardial I/R	RIPC; NaHS or L-cysteine; HIF-1α inhibitor YC-1	H₂S-related intervention restored HIF-1α and Nrf2 nuclear translocation; YC-1 reduced Nrf2 nuclear accumulation and cardioprotection	H₂S/RIPC → HIF-1α → Nrf2 nuclear recruitment	Direct MIRI-context evidence; HIF-1α inhibition reduced Nrf2 nuclear recruitment and cardioprotection after RIPC.
Evidence for Nrf2 → HIF-1α regulation					
(128)	Breast cancer cells; HIF1A enhancer-regulation model	Nrf2/ARE modulation; ChIP and reporter assays; CRISPR editing of the HIF1A enhancer	ARE disruption reduced Nrf2 binding, HIF1A mRNA, HIF-1α protein, and downstream target-gene expression	Nrf2–sMaf → HIF1A cis-regulatory ARE (∼32 kb upstream) → HIF1A transcription	Strong direct genomic evidence; non-MIRI model establishes Nrf2→HIF1A transcription, requiring myocardial validation.
(129)	Breast cancer cells; hypoxia	Nrf2 silencing; miR-181c OE/inhibition	Nrf2 silencing reduced HIF-1α and glycolytic target genes; miR-181c inhibition partially rescued the effect	Nrf2 ⊣ miR-181c → HIF-1α accumulation and metabolic transcription	Causal rescue evidence under hypoxia; supports a transferable miR-181c relay, pending myocardial I/R validation.
(130)	HCC cell/liver models	Nrf2 hyperactivation or loss; protein-interaction and PHD2/VHL analyses	Nrf2 promoted HIF-1α protein stability, nuclear accumulation, and transcriptional activity	Direct Nrf2 binding to HIF-1α ODD → PHD2 access/hydroxylation ↓ → VHL-dependent degradation ↓	Strong direct protein-stability evidence; non-MIRI model establishes Nrf2–HIF-1α/PHD2–VHL regulation.
(131)	Tumor and related cell lines; protein-stability models	NQO1 KD/OE; protein-binding, ubiquitination, and half-life assays	NQO1 prolonged HIF-1α half-life and enhanced HIF signaling	Nrf2 → NQO1 → PHD–HIF-1α interaction ↓ → proteasomal degradation ↓	Effector-level mechanistic evidence; supports NQO1-mediated HIF-1α stabilization, pending MIRI validation.
(133)	Renal tubular epithelial cells; hypoxia	Nrf2 deficiency/inhibition; HIF1A/HIF-1α and HIF target-gene measurements	Nrf2 loss attenuated HIF1A expression, HIF-1α activation, and HIF-dependent metabolic transcription	Nrf2-dependent HIF1A transcriptional supply → HIF-1α activation	Hypoxia-context causal evidence; supports Nrf2-dependent HIF1A/HIF-1α regulation under oxygen stress.
(135)	A549 lung adenocarcinoma cells; intermittent hypoxia	NOX1/Nrf2/Trx1-related interventions	Nrf2-dependent Trx1 induction enhanced HIF-1α accumulation and HIF-dependent transcription	NOX1/ROS → Nrf2 → Trx1 → HIF-1α	Redox-adaptation evidence; supports an Nrf2–Trx1–HIF-1α relay relevant to preadaptation, but not myocardial I/R.
Evidence for shared/reciprocal proteostasis regulation					
(134)	Cell lines; proteostasis model	UBXN7-related genetic/molecular manipulation	UBXN7 manipulation altered both Nrf2 and HIF-1α protein abundance and cellular metabolic state	UBXN7 links CRL3–Keap1 and CRL2–VHL ubiquitin-ligase systems → coordinated Nrf2/HIF-1α homeostasis	Shared-proteostasis evidence; identifies a reciprocal regulatory interface whose role in MIRI remains untested.

Non-MIRI studies were included when they provided causal evidence for conserved hypoxia/reoxygenation- or redox-responsive mechanisms relevant to HIF-1α–Nrf2 regulation. Evidence from cardiac, I/R, H/R, or OGD/R models was considered to have greater pathophysiological proximity to MIRI. These studies support mechanistic inference and biological plausibility, but do not substitute for direct validation in myocardial I/R models.

↑, increase/activation; ↓, decrease/inhibition; ⊣, negative regulation; ARE, antioxidant response element; ChIP, chromatin immunoprecipitation; CRISPR, clustered regularly interspaced short palindromic repeats; FPN1, ferroportin 1; HCC, hepatocellular carcinoma; H₂S, hydrogen sulfide; HRE, hypoxia-response element; I/R, ischemia/reperfusion; IRI, ischemia–reperfusion injury; KD, knockdown; MIRI, myocardial ischemia–reperfusion injury; NQO1, NAD(P)H quinone oxidoreductase 1; ODD, oxygen-dependent degradation domain; OE, overexpression; OGD/R, oxygen-glucose deprivation/reoxygenation; PHD, prolyl hydroxylase domain protein; RIPC, remote ischemic preconditioning; ROS, reactive oxygen species; Trx1, thioredoxin 1; UCP2, uncoupling protein 2; VHL, von Hippel–Lindau protein.

## 6 Preconditioning evidence

In myocardial I/R models, several studies using preconditioning paradigms have provided relatively direct evidence that HIF-1*α* can recruit Nrf2-dependent antioxidant responses during preconditioning. Dihydrotanshinone I transiently inhibits mitochondrial respiratory-chain complex I, generating a moderate redox signal that stabilizes HIF-1*α*, which subsequently increases Nrf2 promoter activity, transcription, and nuclear accumulation and upregulates antioxidant genes such as HO-1 and NQO1. HIF-1*α* knockdown markedly attenuates both Nrf2 activation and the associated cardioprotective effects (118). Notably, this protection also exhibits a clear sequence dependence. Administration of dihydrotanshinone I to initiate HIF-1*α*-mediated adaptation before protocatechualdehyde-mediated expansion of reducing reserves such as GSH provides greater protection than the reverse sequence; a similar pattern is observed when metformin precedes vitamin E (118). These findings suggest that the efficacy of preconditioning may depend not only on whether the relevant protective pathways are activated, but also on whether metabolic adaptation and antioxidant defense are established in an appropriate temporal sequence. In aged rats subjected to RIPC followed by myocardial I/R, NaHS and L-cysteine restore attenuated HIF-1*α* expression, Nrf2 nuclear translocation, and cardioprotection, whereas the HIF-1*α* inhibitor YC-1 reduces Nrf2 nuclear accumulation and protective efficacy, further supporting HIF-1*α*-dependent recruitment of Nrf2 during preconditioning (136).

Beyond direct myocardial I/R preconditioning studies, other pharmacological and non-pharmacological interventions further indicate that HIF-1*α*–Nrf2-related protective programs are amenable to exogenous modulation. In diabetic myocardial injury, roxadustat stabilizes HIF-1*α*, increases UCP2 expression, and enhances PI3 K/Akt and Nrf2-related antioxidant responses, whereas loss of HIF-1*α* or UCP2 markedly attenuates these effects (121). Supportive evidence from non-cardiac models shows that combined salvianolic acid B and benzoylpaeoniflorin increases Nrf2/HO-1, HIF-1*α*, and VEGFA expression and promotes angiogenesis in experimental ischemic stroke and endothelial oxygen–glucose deprivation/reoxygenation models; Nrf2 inhibition with ML385 attenuates Nrf2/HO-1/VEGFA signaling and the pro-angiogenic effect (137). In hypoxic cardiomyocytes, baicalin enhances Nrf2/HO-1 signaling together with HIF-1*α*- and BNIP3-related mitochondrial protective responses, thereby reducing mitochondrial dysfunction and cellular injury (138). Non-pharmacological interventions show similar regulatory potential. In diabetic cardiomyopathy, long-term aerobic exercise promotes HIF-1*α* stabilization and enhances Nrf2/GPX4-dependent antioxidant defense, whereas HIF-1*α* knockdown attenuates exercise-induced Nrf2 activation and anti-ferroptotic effects (139). Although these studies were not all performed using acute MIRI preconditioning paradigms, they collectively indicate that HIF-1*α*- and Nrf2-related adaptive and antioxidant programs can be experimentally modulated and recruited in advance by pharmacological or non-pharmacological interventions.

The importance of these intervention studies lies in showing that HIF-1*α*–Nrf2 crosstalk may not only be mechanistically plausible but may also provide an actionable window for therapeutic modulation. In particular, the sequence dependence observed in preconditioning studies suggests that protective efficacy may depend on the timing and duration of recruitment of distinct adaptive programs rather than simply on the magnitude of single-pathway activation. Compared with enhancement of a single pathway, targeting HIF-1*α*–Nrf2-related regulation may simultaneously reduce oxidative susceptibility before reoxygenation and increase myocardial tolerance to oxidative stress during reperfusion, thereby providing a rationale for temporally coordinated interventions spanning the continuous pathological course of MIRI. Representative preconditioning and preadaptation interventions involving HIF-1*α*–Nrf2-related signaling are summarized in Table 2.

**Table 2:** Preconditioning and related interventions involving HIF-1α–Nrf2 signaling.

Reference	Intervention / paradigm	Model / context	Main HIF-1α/Nrf2-related finding	Relevance to MIRI
A. Direct myocardial I/R preconditioning evidence				
(118)	Sequential preconditioning: dihydrotanshinone I → protocatechualdehyde; metformin → vitamin E	Myocardial I/R preconditioning models	Early HIF-1α stabilization induced Nrf2 transcription, nuclear accumulation, and antioxidant gene expression. Protection was greatest when HIF-1α activation preceded antioxidant supplementation.	Relatively direct evidence. HIF-1α knockdown reduced Nrf2 activation and cardioprotection; sequence comparisons supported the proposed temporal order.
(136)	RIPC with NaHS or L-cysteine	Aged rats: RIPC before myocardial I/R	NaHS or L-cysteine restored blunted HIF-1α expression, Nrf2 nuclear translocation, and cardioprotection; YC-1 attenuated these effects.	Relatively direct evidence that HIF-1α-dependent Nrf2 recruitment contributes to RIPC protection.
B. Supporting evidence from cardiac and non-cardiac models				
(121)	Roxadustat	Diabetic myocardial injury and high-glucose cardiac models	HIF-1α stabilization increased UCP2 and PI3K/Akt- and Nrf2-related antioxidant responses; loss of HIF-1α or UCP2 attenuated these effects.	Supportive cardiac evidence; not an acute MIRI preconditioning study.
(137)	Salvianolic acid B+benzoylpaeoniflorin	MCAO rats, ischemic zebrafish, and OGD/R-treated HUVECs	Combined treatment increased Nrf2, HO-1, HIF-1α, and VEGFA expression and promoted angiogenesis; ML385 reduced Nrf2/HO-1/VEGFA activation and the pro-angiogenic effect.	Supportive evidence from non-cardiac ischemia models; not a cardiac preconditioning study.
(138)	Baicalin	Hypoxic cardiomyocytes	Activated Nrf2/HO-1 together with HIF-1α/BNIP3-related mitochondrial protection, reducing mitochondrial dysfunction and cell injury.	Supportive cardiac-cell evidence; not a MIRI preconditioning model.
(139)	Long-term aerobic exercise	Diabetic cardiomyopathy	Stabilized HIF-1α and enhanced Nrf2/GPX4-dependent antioxidant defense; HIF-1α knockdown reduced Nrf2 activation and anti-ferroptotic effects.	Supportive cardiac pre-adaptation evidence; not acute MIRI.

Section A summarizes studies providing relatively direct evidence for HIF-1α–Nrf2-related regulation in myocardial I/R preconditioning settings. Section B includes supportive cardiac intervention or pre-adaptation studies showing that HIF-1α- and Nrf2-related protective programs can be experimentally modulated, but these should not be interpreted as direct validation of a stable bidirectional regulatory network in MIRI.

HUVECs, human umbilical vein endothelial cells; I/R, ischemia/reperfusion; MCAO, middle cerebral artery occlusion; MIRI, myocardial ischemia–reperfusion injury; NaHS, sodium hydrosulfide; OGD/R, oxygen–glucose deprivation/reoxygenation; RIPC, remote ischemic preconditioning.

## 7 Evidence boundaries and future directions

### 7.1 Limitations of the current evidence

The intervention studies summarized above support the potential controllability of HIF-1*α*–Nrf2-related regulation, but do not establish a stable bidirectional regulatory network in MIRI. Evidence for the effects of Nrf2 on HIF-1*α* is available at multiple levels, including *HIF1A* transcription, post-transcriptional regulation, and HIF-1*α* protein stability. By contrast, regulation of Nrf2 by HIF-1*α* is supported mainly by changes in Nrf2 expression, nuclear translocation, and transcriptional output after HIF-1*α* inhibition or silencing, together with candidate relay mechanisms such as FPN1 and UCP2-PI3K/Akt; direct mechanistic evidence remains comparatively limited. Moreover, direct myocardial I/R evidence is concentrated largely on HIF-1*α*-dependent recruitment of Nrf2 during preconditioning, whereas much of the evidence supporting reciprocal regulation derives from other cardiac conditions or non-cardiac hypoxia, I/R, and redox-stress models.

Simultaneous changes in HIF-1*α* and Nrf2 also do not by themselves establish regulatory coupling, because hypoxia, ROS, metabolic stress, and other shared upstream stimuli may activate both pathways in parallel. Studies incorporating reciprocal intervention, functional rescue, and validation of cardioprotective outcomes within a unified MIRI system remain scarce. At present, the HIF-1*α*–Nrf2 axis is therefore more appropriately regarded as a biologically plausible potential regulatory network whose directionality, temporal organization, and integrated cardioprotective effects require further validation in standardized MIRI models.

### 7.2 Translational considerations

The potential clinical application of HIF-1*α*–Nrf2-related regulation will depend strongly on whether the intervention can be matched to an available ischemia–reperfusion window. In acute myocardial infarction, ischemia has already begun before medical treatment is initiated, making classical preconditioning before ischemia difficult to implement. A more practical window may lie during ongoing ischemia and before reperfusion, when remote ischemic periconditioning or rapidly acting pharmacological interventions could modify the myocardial adaptive state before coronary flow is restored (48, 50). By contrast, cardiac surgery provides a predictable ischemia–reperfusion window in which preconditioning can be applied before myocardial ischemia (9). This setting may therefore be more suitable for testing whether HIF-1*α*-mediated metabolic adaptation can be established before ischemia and Nrf2-dependent antioxidant defense reinforced before reperfusion in an appropriately ordered manner.

Clinical experience with ischemic conditioning nevertheless indicates that an accessible intervention window alone is insufficient to ensure cardioprotection. In acute myocardial infarction treated with primary percutaneous coronary intervention, the large CONDI-2/ERIC-PPCI trial found no reduction in cardiac death or hospitalization for heart failure after remote ischemic conditioning (140). Similarly, despite the predictable intervention window of cardiac surgery, the ERICCA and RIPHeart trials did not demonstrate significant improvements in major clinical outcomes after remote ischemic preconditioning (141, 142). These trials were not designed to test HIF-1*α*–Nrf2 regulation specifically, but they illustrate the broader difficulty of translating experimental conditioning into reproducible clinical benefit. Effective translation will therefore require not only an appropriate intervention window, but also sufficient and appropriately timed engagement of the intended protective programs.

The magnitude and duration of pathway activation are equally important. The objective should not be sustained enhancement of either HIF-1*α* or Nrf2, but transient and reversible recruitment of protective programs within the relevant stage of MIRI. Prolonged HIF-1*α* activity may maintain glycolytic dependence, delay recovery of mitochondrial oxidative metabolism, and disrupt organelle homeostasis (87–92). Nrf2 activation is likewise context dependent, and sustained or excessive activation cannot be assumed to remain uniformly protective outside the acute redox-stress setting (130). Pharmacological modulation must therefore balance dose, duration of action, reversibility, and tissue specificity. Age, diabetes, metabolic disorders, concomitant medications, and perioperative conditions may further alter pathway responsiveness and contribute to differences between controlled experimental models and clinical populations (7–9, 49). Thus, clinical translation is likely to depend on transient, stage-appropriate, and temporally coordinated regulation rather than maximal or sustained activation of either pathway.

### 7.3 Future directions

Future studies should first establish causal evidence for reciprocal regulation between HIF-1*α* and Nrf2 within a unified MIRI model. Gene silencing or knockout, pharmacological blockade, and functional rescue should be applied in both regulatory directions to determine how HIF-1*α* affects Nrf2 expression, nuclear translocation, and ARE-dependent transcription, and how Nrf2 affects *HIF1A* transcription, HIF-1*α* protein stability, and HRE-dependent output. Parallel activation driven by common upstream stimuli should also be excluded. Protective effects should then be compared across isolated HIF-1*α* or Nrf2 modulation, combined modulation, sequential modulation, and dual blockade to determine whether the two pathways exhibit directional signal transmission and whether coordinated regulation provides protection beyond single-pathway intervention.

Assessment of the proposed network should also move beyond static expression measurements toward time-resolved functional evaluation. HIF-1*α* stabilization and HRE-dependent transcriptional output, Nrf2 nuclear translocation and ARE-dependent transcriptional activity, and candidate relay components such as FPN1 and UCP2-PI3 K/Akt should be monitored across preconditioning, ischemia, and early reperfusion, together with energy metabolism, mitochondrial function, redox status, and myocardial injury outcomes. Evidence for functional coupling would be strengthened if the two pathways change in a reproducible temporal sequence and perturbation of one node interrupts the functional output and corresponding protective effect of the other. On this basis, combinations of markers reflecting pathway activation, signal coupling, and protective efficacy could be identified to establish a dynamic framework for evaluating HIF-1*α*–Nrf2 regulation.

Once causal coupling and its temporal organization have been established, candidate interventions should be evaluated in models that more closely reproduce the clinical settings described above. Particular attention should be given to whether sequential or coordinated modulation provides additional protection over single-pathway intervention and whether the corresponding pathway responses can be captured by reproducible target-engagement markers. Incorporating such markers together with myocardial injury and functional outcomes would provide a more direct basis for subsequent translational studies and allow the therapeutic potential of HIF-1*α*–Nrf2-related regulation to be evaluated without assuming network-level efficacy before its causal basis has been established.

## 8 Conclusions

Taken together, MIRI is best viewed as a temporally continuous process in which metabolic and mitochondrial disturbances established during ischemia shape vulnerability to reperfusion injury. Within this framework, HIF-1*α*-mediated hypoxic/metabolic adaptation and Nrf2-mediated redox defense are functionally complementary, and current evidence supports the possibility of directional crosstalk between them. Preconditioning may exploit this relationship by recruiting adaptive and antioxidant programs before reperfusion, providing a rationale for temporally coordinated rather than single-pathway intervention.

However, the HIF-1*α*–Nrf2 axis cannot yet be considered an established bidirectional regulatory network in MIRI. Direct myocardial I/R evidence remains limited, particularly for HIF-1*α*-to-Nrf2 regulation, and causal validation in unified MIRI models using reciprocal perturbation and functional rescue is still required. Defining the directionality, timing, and target engagement of this interaction will be essential before network-based preconditioning strategies can be meaningfully translated.
